# Supplementary material for: Patients’ experiences and perceptions of physical restraint in psychiatric care: A descriptive qualitative study at Muhimbili National Hospital
Source: PLOS Ment Health. 2026 May 15;3(5):e0000608. doi: 10.1371/journal.pmen.0000608 (PMC13178866; doi:10.1371/journal.pmen.0000608)
Supplement: S1 File — (DOCX) [file pmen.0000608.s001.docx]

**Excerpts from Interviews**

*When a patient is told to sit somewhere and refuses, or hits another patient without cause, that’s when restraints are used so they can be given medication and eventually recover and rejoin others*.” (Participant 12)

*Restraints are used because of human behavior. Some patients are aggressive and refuse treatment. They may need to be restrained to receive medication. It’s for their benefit, to help them get back on track.* (Participant 12)

*If you’re disruptive in the ward, if you don’t settle, while others are resting, they’ll restrain you. … If someone becomes a danger to themselves or others due to lack of calm, they’ll be tied up, … in such cases, it’s necessary to ensure safety.* (Participant 5)

*It's the same reason, they (patients) might be sent home with medication and cause chaos or harm. That creates an emergency and adds to the existing problem. That’s why patients are restrained, to prevent issues in the community.* (Participant 12)

*My legs were swollen from being in one position, and my body felt heavy.* (Participant 8)

*The wrists and ankles get bruised. You can’t move, like being on a cross.* (Participant 7)

*They say it (the restraint) is to prevent the patient from falling after sedation. But I don’t like it. It makes my heart race, causes fatigue, bruising, and lasts long, from noon until the next morning. It’s painful and humiliating*. (Participant 3)

*My stress increased instead of reducing … because I felt I was being restrained for no reason*. (Participant 9)

*I felt like a prisoner, oppressed. It made me question how bad my condition was to deserve that. …Yes, I had nightmares of being crucified. My mom told me it’s because of the restraints.* (Participant 2)

*I felt very bad. I needed to use the toilet but was restrained, so I relieved myself in bed.* (Participant 9)

*It hurts. I feel they (those restrained) are being mistreated. I was once restrained and received an injection, and I defecated on myself. It was very embarrassing.* (Participant 11)

*I was tied, ... They should have explained to me instead of tying me up and walking me in public like that*. (Participant 3)

*It was at night. We’d taken our sleeping medication but hadn’t slept. We were chatting in one bed. Staff came in and tied us up, probably to force us to sleep*. (Participant 5)

*I felt unsupported. They tied us and left to sleep. Even when I cried out in pain, no one came. ... I felt very lonely. I kept calling for help but no one came. I felt discouraged.* (Participant 5)

*I called the nurse and asked to be released to use the toilet, but they didn’t come.* (Participant 10)

*No one asked me about my feelings. … It creates a bit of a gap. No one came to ask about my emotions, it becomes difficult.* (Participant 12)

*I thought they would explain what happened and what to expect. That would have helped me understand better*. (Participant 4)

*Being restrained feels like a punishment. When your mental state returns, the pain is intense, and it acts as a deterrent, you’ll want to avoid being restrained again*. … When restrained, you feel like a victim, mistreated and misunderstood (Participant 6)

*I don’t believe it's the only solution. They should find other ways. It's not necessary to tie someone up. … tying was unwarranted. It sends the wrong message.* (Participant 3)

*It (restraint) is not a crime. The hospital is a place for care and discipline*. (Participant 10)

*To assess their mental condition, control their aggression, and monitor their behavior.* (Participant 12)

*You lose trust and start fearing the caregivers after being restrained. … Yes, I lost trust and started strictly following instructions to avoid being tied again.* (Participant 5)

*They should know that being restrained puts you in a terrible state. You lose trust and start fearing the caregivers.* (Participant 5)

*It’s hard. You develop some resentment. They say it was for your safety, and you forgive them. But it still hurts.* (Participant 1)

*At first, I felt mistreated, but later I understood, it’s part of the process. If you’re calm, they help you*. (Participant 10)

*Don’t take things lightly. Be respectful and gentle with patients. Don’t restrain unnecessarily, it may drive patients away. … Be honest and explain the situation to the patient, ask for their cooperation, like a police officer would.* (Participant 10)

*Let patients rest instead of restraining. Use sleeping pills or injections to calm them. Tying should be a last resort.* (Participant 2)

*If someone has a problem, talk to them. There’s no need to tie them.* (Participant 2)

*They should listen to the patient, hear what they need and give advice if it's not harmful.* (Participant 9)

*A special room with nothing harmful inside, where the patient can be observed until calm and then returned to the ward*. (Participant 1)

*If you must restrain, do it gently, don’t cause injury. Blood needs to circulate properly. Restrain moderately and communicate.* (Participant 11)

*Use restraint only for those with aggressive behavior, and don’t keep them tied for too long.* (Participant 10)
