# Supplementary material for: Patients’ experiences and perceptions of physical restraint in psychiatric care: A descriptive qualitative study at Muhimbili National Hospital
Source: PLOS Ment Health. 2026 May 15;3(5):e0000608. doi: 10.1371/journal.pmen.0000608 (PMC13178866; doi:10.1371/journal.pmen.0000608)
Supplement: Attachment — Submitted filename: Response to Reviewers.docx [file pmen.0000608.s002.docx]

**Patients’ Experiences and Perceptions of Physical Restraint in Psychiatric Care. A Descriptive Qualitative Study at Muhimbili National Hospital**

We would like to thank the reviewer for the comments provided to improve this study. We have revised the manuscript as shown in the point-to-point responses to reviewer’s comments provided in the table below.

| **Comments** | **Authors’ Response** |
| --- | --- |
| **Journal requirements/Editor’s Comments:** |  |
| 1. Please describe in your methods section how capacity to provide consent was determined for the participants in this study. Please also state whether your ethics committee or IRB approved this consent procedure. If you did not assess capacity to consent please briefly outline why this was not necessary in this case. | Thank you for your comment regarding how participants’ capacity to provide informed consent was determined. We assessed participants’ decision-making capacity using a mental status evaluation to determine their ability to provide informed consent and engage meaningfully in the interview. This procedure was indicated in the data collection subsection of the Methods; however, we have now added further details to clarify what the mental status evaluation entailed. This procedure was included in the study proposal that was approved by the Research Ethics Committee. |
| 2. We note that your Data Availability Statement is currently as follows: "All relevant data are within the manuscript and its Supporting Information files."  Please confirm at this time whether or not your submission contains all raw data required to replicate the results of your study. Authors must share the “minimal data set” for their submission. PLOS defines the minimal data set to consist of the data required to replicate all study findings reported in the article, as well as related metadata and methods (https://journals.plos.org/mentalhealth/s/data-availability#loc-minimal-data-set-definition). | Thank you for this comment. We confirm that all relevant data are available within the manuscript and its Supporting Information files. Excerpts from the in-depth interviews used to generate the illustrative quotations have been provided as a Supporting Information file. |
| 1. If the reviewer comments include a recommendation to cite specific previously published works, please review and evaluate these publications to determine whether they are relevant and should be cited. There is no requirement to cite these works unless the editor has indicated otherwise. | Thank you for this comment. There are some recommendations from the reviewer to cite specific previously published works. Their relevance has been determined before deciding to use them. |
| **Reviewer #1** |  |
| Thank you for the opportunity to review this manuscript. It is well-written and addresses an important topic that has received limited attention in the study context, making this contribution particularly relevant and timely.  Here are areas for improvement in the manuscript. | Thank you for your positive and encouraging feedback. We sincerely appreciate your recognition of the manuscript’s relevance, clarity, and contribution to an underexplored but important topic within the study context. Your comments affirm the significance of this work, and we are grateful for your thoughtful review and support. |
| 1. Page 3, Line 45: How would the authors differentiate the definition of physical restraint from mechanical restraint? | Thank you for this important comment. In the context of this study, physical restraint was used as an umbrella term referring to any manual or device-assisted method used to restrict a patient’s movement to prevent harm to self or others. However, we acknowledge the need to distinguish this from mechanical restraint. Mechanical restraint specifically refers to the use of devices or equipment (e.g., straps, belts, or cloth ties) to limit a patient’s movement, whereas physical restraint may also include manual holding by staff without the use of devices. We have made a revision to clarify this difference |
| 2. Lines 58-60: More recent studies estimate the global prevalence of restrictive measures. See Belayneh et al., 2024; Savage et al., 2024. | Thank you for this observation. We have revised the estimates of the global prevalence of restrictive measures using more recent studies, as suggested |
| 3. For a study conducted in a low-resource setting, the authors do not engage sufficiently with literature from low-resource settings. Given the limited number of qualitative studies on this theme conducted on the African continent, including Nigeria and Ghana, I expect them all to be referenced in the manuscript. | Thank you for this important observation. We have revised the Introduction to incorporate evidence from low-resource settings, with particular emphasis on qualitative studies conducted in African contexts. Relevant literature from countries such as Nigeria, Ghana, and Ethiopia have been included to better situate the study within the regional evidence base and to strengthen the contextual relevance of the research. |
| 4. Other countries on the continent have explored the experiences and perceptions of patients regarding coercive measures, including physical restraints (see Aluh et al., 2022). How will the current study differ from what has been reported? Consider providing a brief description of the unique context in Tanzania and how it differs from other African countries to give readers a clearer understanding. | Although studies from other African countries, such as Nigeria and Ghana, have explored service users’ experiences of coercive measures, these findings may not be transferable to Tanzania due to differences in mental health service organization, resource availability, cultural norms, and the implementation of mental health policies. This information has been added to the introduction section. |
| 5. There should be a description of the authors` positionality and reflexivity. Is the P.I. a staff member of the same hospital? | Thank you for this important comment. We have added a description of PI’s positionality and reflexivity in the data collection subsection of the Methods section. The manuscript now clarifies the researcher’s professional background, relationship to the study setting, and the steps taken to minimize potential bias during data collection and analysis. |
| 6. Which ethical safeguards were in place to manage the power dynamics between the P.I. and the study participants? | Thank you for this important comment. We have revised the Ethical Considerations section to clarify the safeguards implemented to minimize potential power imbalances between the principal investigator and participants. The manuscript now describes measures such as voluntary recruitment, the absence of a clinical care relationship, emphasis on the right to decline or withdraw without consequences, and the use of a private, non-clinical interview setting to promote openness and reduce perceived coercion. |
| 7. In the abstract, it is written that a framework method of analysis was used, and in the manuscript, a thematic analysis was used. Please clarify. | Thank you for pointing this out. We apologize for the inconsistency. The study used thematic analysis for data analysis, following Braun and Clarke’s six-step framework. The abstract has been revised to accurately reflect this. |
| 8. Given that the study aims to explore these experiences in order to provide context-specific recommendations, the recommendation section is not sufficiently explored. A description of the Tanzanian context would allow readers to understand how and why physical restraints are used. Key Contextual Questions to Address include: What is the legal framework? Is there a national mental health Act regulating coercive measures? Is Tanzania a signatory to the UNCRPD, the African Charter on Human and Peoples' Rights (ACHPR), and its disability protocol? How many mental health services and professionals are in the country? Do patients pay out-of-pocket for mental health care? Is there a cultural connotation to the use and acceptance of physical restraints by professionals and patients? Introduce these details in the background section, then link them with the results in the discussion to make the manuscript richer and more nuanced. | Thank you for this insightful comment. We have revised the Introduction/Background to incorporate contextual details about Tanzania that help explain the use of physical restraints. The revised text now highlights the Tanzanian mental health landscape, including the existence of the Mental Health Act (2016) regulating coercive measures, Tanzania’s status as a signatory to the UNCRPD, the African Charter on Human and Peoples’ Rights, and the African Disability Protocol, the limited number of mental health services and professionals, reliance on institution-based care with minimal community services, and the economic and cultural factors influencing care delivery. These contextual details have been linked to our study findings in the Discussion to provide a more nuanced interpretation of patients’ experiences and inform context-specific recommendations for improving patient-centered psychiatric care and restraint practices in Tanzania. |
| 9. What are the future research directions on this theme in Tanzanian mental health care and on the continent? | Thank you for this question. We have revised the conclusion to include a statement indicating that future research should focus on evaluating restraint-reduction interventions, examining system and cultural determinants of restraint use, and generating multi-site evidence to inform rights-based, context-appropriate mental health care in Tanzania and other African settings. |
