# Supplementary material for: Patients’ experiences and perceptions of physical restraint in psychiatric care: A descriptive qualitative study at Muhimbili National Hospital
Source: PLOS Ment Health. 2026 May 15;3(5):e0000608. doi: 10.1371/journal.pmen.0000608 (PMC13178866; doi:10.1371/journal.pmen.0000608)
Supplement: Attachment — Submitted filename: Response to Reviewers_2.docx [file pmen.0000608.s003.docx]

**Patients’ Experiences and Perceptions of Physical Restraint in Psychiatric Care. A Descriptive Qualitative Study at Muhimbili National Hospital**

We would like to thank the reviewer for the comments provided to improve this study. We have revised the manuscript as shown in the point-to-point responses to reviewer’s comments provided in the table below.

| **Comments** | **Authors’ Response** |
| --- | --- |
|  |  |
| **Reviewer #2** |  |
| The manuscript presents an interesting, rigorous and well-written study which will add important findings to the literature. I only have minor suggestions for improvement. | We are grateful to the reviewer for the encouraging comments and for recognizing the rigor and contribution of our work. We have carefully considered the minor suggestions and have addressed them accordingly to further improve the manuscript. |
| **Abstract:** |  |
| It would be helpful to include the number of study participants. | We thank the reviewer for this helpful suggestion. We have now clarified the number of study participants (14). |
| **Introduction:** |  |
| l. 35: What does “the prevalence of physical restraint among adult psychiatric inpatients” refer to? E.g., prevalence of experiencing restraint at least once during their stay? | We thank the reviewer for this important clarification. In our manuscript, ‘the prevalence of physical restraint among adult psychiatric inpatients’ refers to the proportion of patients who were exposed to at least one form of restrictive care practice during the period in which they received inpatient mental health services. We have now clarified this definition in the introduction. |
| **Methods:** |  |
| l. 169: “recruitment was conducted through clinic staff rather than direct solicitation” - It would be helpful to explain how exactly participants were recruited, e.g. who exactly approached them about participation (treating physicians?) and how/when this was done, and whether participants received any form of compensation for participation in the study. | Thank you for this comment. We have revised the Data Collection subsection to clarify how participants were recruited, including who approached them, how and when this occurred, and that participants did not receive any form of compensation. |
| Regarding reflexivity, it would be helpful to add a statement on the researchers’ own position regarding the use of coercion in mental healthcare, and how this might have influenced the study results. | We thank the reviewer for this suggestion. We have added a statement on reflexivity clarifying the researchers’ position. As supported by research evidence, the research team holds the position that less restrictive measures should be prioritized in mental healthcare. |
| l.111: It would be helpful to explain the term “Vikuruti rehabilitation” for international readers | Thank you for this suggestion. We have added an explanation of the term ‘Vikuruti rehabilitation’ for international readers, clarifying that it refers to a structured psychosocial rehabilitation center for patients recovering from mental health crises. |
| **Results:** |  |
| I worry whether the detailed description of participants including exact ages, exact treatment duration and occupation risks re-identification of individual patients. I would rather include age ranges (e.g., 20-30 etc.) and ranges of treatment duration, and I am not sure whether information about occupation is relevant to the study – especially given that the text states that most “reported informal employment or were unemployed”. (This seems like a discrepancy between employment information in the table and in the text?) In addition, could the authors provide information about participants’ diagnoses, e.g. bipolar / schizophrenia / etc? | We thank the reviewer for this important comment. To protect participant confidentiality and reduce the risk of re-identification, we have revised Table 1 and the text to present age ranges (e.g., 20–30, 31–40, etc.) and ranges for treatment duration rather than exact values. Regarding occupation, we have simplified the text to focus on broad categories (e.g., employed in formal/informal work), as detailed occupational information is not central to the study objectives. Additionally, we have included participants’ primary psychiatric diagnoses in the revised text to provide relevant clinical context. The table and text have been updated to ensure consistency and maintain participant anonymity while conveying necessary socio-demographic and clinical information |
| Table 2 is somewhat hard to read. I recommend creating individual rows for the subthemes of each theme, rather than separating subthemes by semicolons. It is unclear which subthemes the illustrative codes correspond to. It would be helpful to either align illustrative codes with the corresponding subthemes or to leave them out. | We thank the reviewer for this suggestion. We have revised Table 2 to create individual rows for each subtheme under the main themes for improved readability. Following the reviewer’s recommendation, we have removed the illustrative codes to avoid confusion and make the table clearer. |
| **Discussion:** |  |
| l. 477ff. Patients in this study mentioned several alternatives to restraint, including isolation, observation, and forms of informal coercion. It might be helpful to discuss that these interventions are also perceived as coercive by many patients, so do not necessarily constitute a benign intervention from the perspective of all patients. E.g., the authors might reference literature on informal coercion, and literature on how patients may have different perceptions of which interventions is considered the least restrictive. | We thank the reviewer for this insightful comment. We agree that alternatives to physical restraint, such as seclusion, observation, and informal coercion, may also be experienced as coercive by patients and are not universally perceived as benign. We have revised the Discussion to reflect this nuance and have incorporated relevant literature on informal coercion and patient perceptions of restrictive interventions. |
| Typos/language:  l. 69: Typo: “Evidence from African context is more limited”  l. 76-77: “Patients’ experiences and perceptions regarding coercive practices remain underexplored across African”  l. 277: “being treated like a child despite adulthood” – I would recommend removing “despite adulthood”, as this is clear from the context  l. 417: “as reported by a male” – I would add “participant” here | Thank you for noting this typographical issue. We have corrected all the sentences for grammatical accuracy as suggested. |
